# Synthetic molecular sensors based on CRISPR‐Cas9 redirect anticancer signal flows to treat retinoblastomas

**DOI:** 10.1002/ctm2.618

**Published:** 2021-11-08

**Authors:** Lulu Xiao, Jun Li, Yiyu Sheng, Ye Wang, Xiaoyan Dou

**Affiliations:** ^1^ Department of Ophthalmology Shenzhen Hospital of Southern Medical University Southern Medical University Shenzhen China; ^2^ The Seventh Affiliated Hospital of Sun Yat‐sen University Shenzhen China; ^3^ Department of Ophthalmology Shenzhen Second People′s Hospital The First Affiliated Hospital of Shenzhen University Shenzhen China

Dear editor:

The abnormality of intracellular cancer‐related signal pathways is a critical factor for the emergence of retinoblastoma (RB).^1^ Herein, we developed molecular sensors based on CRISPR‐dCas9^2^ that acted on RB cells, which aimed to redirect the signaling pathways in RB cells, and to induce tumor cell apoptosis and inhibit the malignant phenotype of these cells.

The dCas9‐based molecular sensor acted as the center of intracellular reprogrammed signaling pathways to control endogenous proteins. The single guiding RNA (sgRNA) was engineered by integrating aptamers^3^ that responded to signals and ribozymes involved in self‐cleavage, thus linking intracellular signals to gene expression events (Figure 1). The aptamer of tetracycline was combined with hammerhead ribozyme sequences as additional sequences of sgRNA to reprogram the CRISPR‐dCas9 system and construct corresponding activating (tet‐activator) and inhibitory (tet‐inhibitor) sensors. The fluorescence expressions in HEK293t cells were used to evaluate the inhibitory and activation effects, respectively, of the sensors. We used different tetracycline concentrations as the experimental control groups, and found that the degrees of activation of tet‐activators (Figure 1) and inhibition of tet‐inhibitors (Figure 1) were tetracycline concentration dependent. In addition, the mutant aptamers were used to replace regions of the aptamers to construct negative sgRNAs and the unmodified wild‐type sgRNAs as experimental control groups. We found that the experimental results of negative sgRNA groups were not significantly different from the activation and inhibition effects of tet‐sensors at a tetracycline concentration of 0 μmol/L, which indicated that tet‐sensors had an excellent silencing effect without target signal molecules. However, WT‐sgRNA showed better output effects than tet‐sensors using all concentrations of tetracycline, which indicated that the design of molecular sensors may have had a certain impact on the function of the CRISPR‐dCas9 system. Next, we tested the effectiveness of synthetic molecular sensors on endogenous genes such as vascular endothelial growth factor (VEGF) in HEK‐293t cells (Figure 1J). After adding tetracycline, the VEGF expressions were increased and decreased under the action of tet‐activators and tet‐inhibitors, respectively (Figure 1).

**FIGURE 1:**
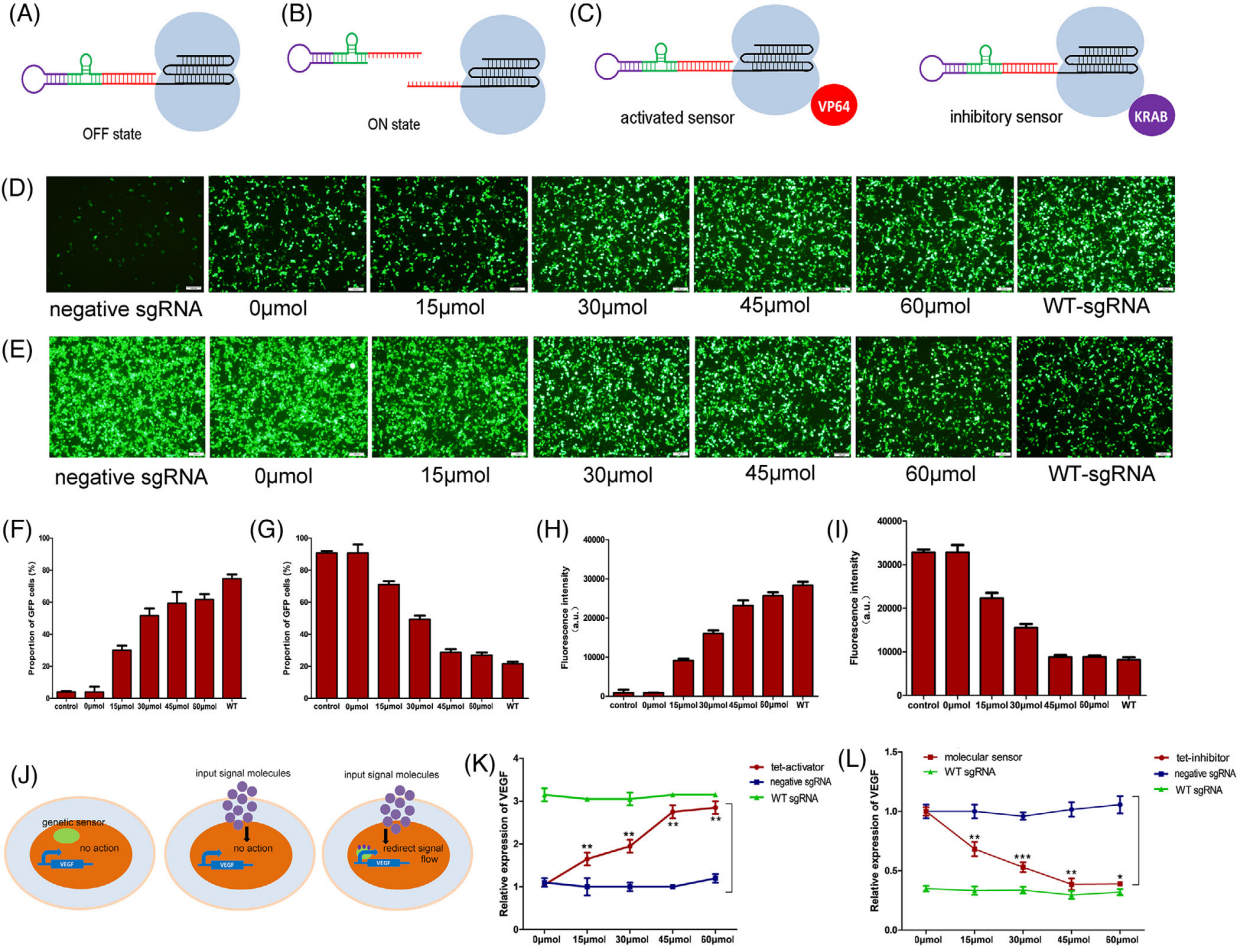
Design and effectiveness of the molecular sensor; the aptamer was combined with the ribozyme to construct a riboswitch, and the riboswitch was connected to the 5′ end of the single guiding RNA (sgRNA). (A) The spacer region of sgRNA bound to the riboswitch sequence, which is in the “off” state; (B) the aptamer part of the riboswitch specifically binds to the target input molecules, which undergoes a conformational change. The riboswitch was separated from the sgRNA, and the sgRNA was switched to the “on” state. (C) The figure on the left shows the activated sensor, and the figure on the right shows the inhibitory sensor. Five experimental groups were used with tetracycline concentrations of 0 μmol/L, 15 μmol/L, 30 μmol/L, 45 μmol/L and 60 μmol/L. In addition, wild‐type sgRNA without riboswitch modification was used as the control group. (D) Tet‐activator activates the expression levels of green fluorescent protein (GFP) in mini‐GPF cells under the action of different concentrations of tetracycline. (F) The proportion of GFP‐expressing mini‐GFP cells obtained using flow cytometry, (E) tet‐inhibitor inhibited the expression level of GFP in GPF cells under the action of different concentrations of tetracycline. (G) The proportion of GFP‐expressing GFP cells obtained using flow cytometry, (H & I) verification of the effects of tet‐activator and tet‐inhibitor at different tetracycline concentrations in mini‐luc cells and luc cells, respectively. (J) The molecular sensors regulated the target signal pathways only when the input molecules were present. Five experimental groups were used with different tetracycline concentrations of 0 μmol/L, 15 μmol/L, 30 μmol/L, 45 μmol/L and 60 μmol/L. We also used a mutant aptamer instead of a mutated aptamer to construct a negative sgRNA and used unmodified wild‐type sgRNA as the experimental control group. (K) tet‐activator‐activated vascular endothelial growth factor (VEGF) under the action of different concentrations of tetracycline, (L) tet‐inhibitor‐inhibited VEGF under the action of different concentrations of tetracycline; we repeated each experiment three times (^*^<.05, ^**^<.01, ^***^<.001)

One hypothetical use of molecular sensors is to link oncogenic signals to anticancer pathways. Nucleophosmin (NPM)^3^ serves as a signaling molecule that functions by activating the MAPK pathway during the progression of RB and promotes cell proliferation. Compared to normal cells, NPM was highly expressed in RB cells (Figure 2). We constructed molecular sensors that further reactivated the expression of P53 (TP53) and P21 (CDKN1A), the well‐known tumor suppressors, by sensing intracellular NPM proteins, thereby reconnecting the NPM signaling pathway.^4^ We engineered NPM aptamers^3^ into modular molecular sensors targeting the upstream promoter regions of P53 and P21 in RB cells. The molecular sensors significantly increased the expressions of P53 and P21 compared with the negative control molecular sensor (Figure 2). Cell proliferation of RB cells transfected with molecular sensors was inhibited. In addition, both shRNA‐p53 and shRNA‐p21 partially reversed the inhibition of cell proliferation, indicating that molecular sensors achieved their functions by activating P53 and P21 (Figure 2). The c‐myc is another oncogenic signal protein that activates multiple carcinogenic pathways and promotes RB cell proliferation.^5^ We inserted the NPM aptamer into the sgRNA, which specifically located the upstream region of the c‐myc promoter and expressed dCas9‐KRAB in RB cells and reprogrammed c‐myc signaling‐associated tumor proliferation pathways in response to intracellular NPM. The molecular sensor significantly decreased the expression of c‐myc, in comparison to the negative control sgRNA (Figure 2). As expected, upregulating the expression of c‐myc partially reversed the inhibitory effect of cell growth by molecular sensors, suggesting that molecular sensors performed their functions by inhibiting c‐myc (Figure 2).

**FIGURE 2:**
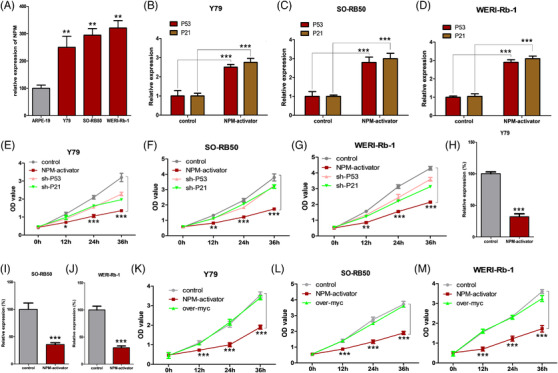
The nucleophosmin (NPM) sensor redirects the effect of anticancer signal flows; construction of molecular sensors that specifically recognized NPMs, and reactivated intracellular P53 and P21 related tumor suppressor pathways by recognizing NPM molecules within retinoblastoma (RB) cells. NPM molecules in RB cells inhibited intracellular c‐myc‐related cancer‐promoting pathways. (A) The expressions of NPM in RB cells relative to normal cells, (B, C & D) the activation effect of NPM sensors on P53 and P21 proteins in RB cells, (E, F & G) NPM sensors inhibited the proliferation of RB cells by promoting the expressions of P53 and P21 proteins. (H, I & J) The inhibitory effect of NPM sensor on c‐myc proteins in RB cells, (K, L & M) NPM sensors inhibited the proliferation of RB cells by inhibiting the expression of c‐myc protein; we repeated each experiment three times (^*^<.05, ^**^<.01, ^***^<.001)

We then determined that molecular sensors could redirect the native signal pathways through multiple specific input signals in the corresponding cells to establish an artificial signal network. NF‐κB^6^ and β‐catenin^7^ are important cancer components in the growth of RB cells. For this system, we designed two types of artificial connections based on redirection of signal flows of the two cancer‐related components. The β‐catenin sensor was designed according to our construction protocol, and the aptamer of β‐catenin was inserted at the 5′ end of the sgRNA backbone to sense the input signal of β‐catenin within cells. The spacer region of the β‐catenin sensor was designed to be located in the upstream region of the *Bax* gene promoter. To induce β‐catenin sensors to only trigger the expressions of downstream genes of interest without disturbance from dCas9 and their fusion proteins, the MS2 hairpins^8^ were inserted at the 3′ end of sgRNA, which specifically recruited the MS2‐VP64 fusion proteins (Figure 3). NF‐κB sensors were constructed in the same manner (Figure 3). The spacer region of the NF‐κB sensor was inserted into the promoter region, upstream of the *Bcl2* gene, and the AFP hairpin^8^ was inserted at the 3′ end of the NF‐κB sensor, which specifically recruited AFP‐KRAB protein to inhibit expressions of downstream genes of interest. To handle multiple signals, we constructed an AND‐gate genetic circuit that functioned based on dual‐signal sensing and used NF‐κB and β‐catenin as inputs, which showed a strong output only when both high input expressions were sensed (Figure 3). We then identified the effectiveness of NF‐κB and β‐catenin expression patterns in cancer cells and normal cells (Figure 3), and found that in comparison to the normal cells, the expressions of NF‐κB and β‐catenin were stronger in RB cells. NF‐κB sensors were used to inhibit the expression of the *BCL2* gene, and β‐catenin sensors were used to activate the expression of the *Bax* gene (Figure 3). The dual‐signal genetic circuit based on NF‐κB and β‐catenin molecular sensors selectively triggered cancer cell apoptosis without affecting normal cells (Figure 4).

**FIGURE 3:**
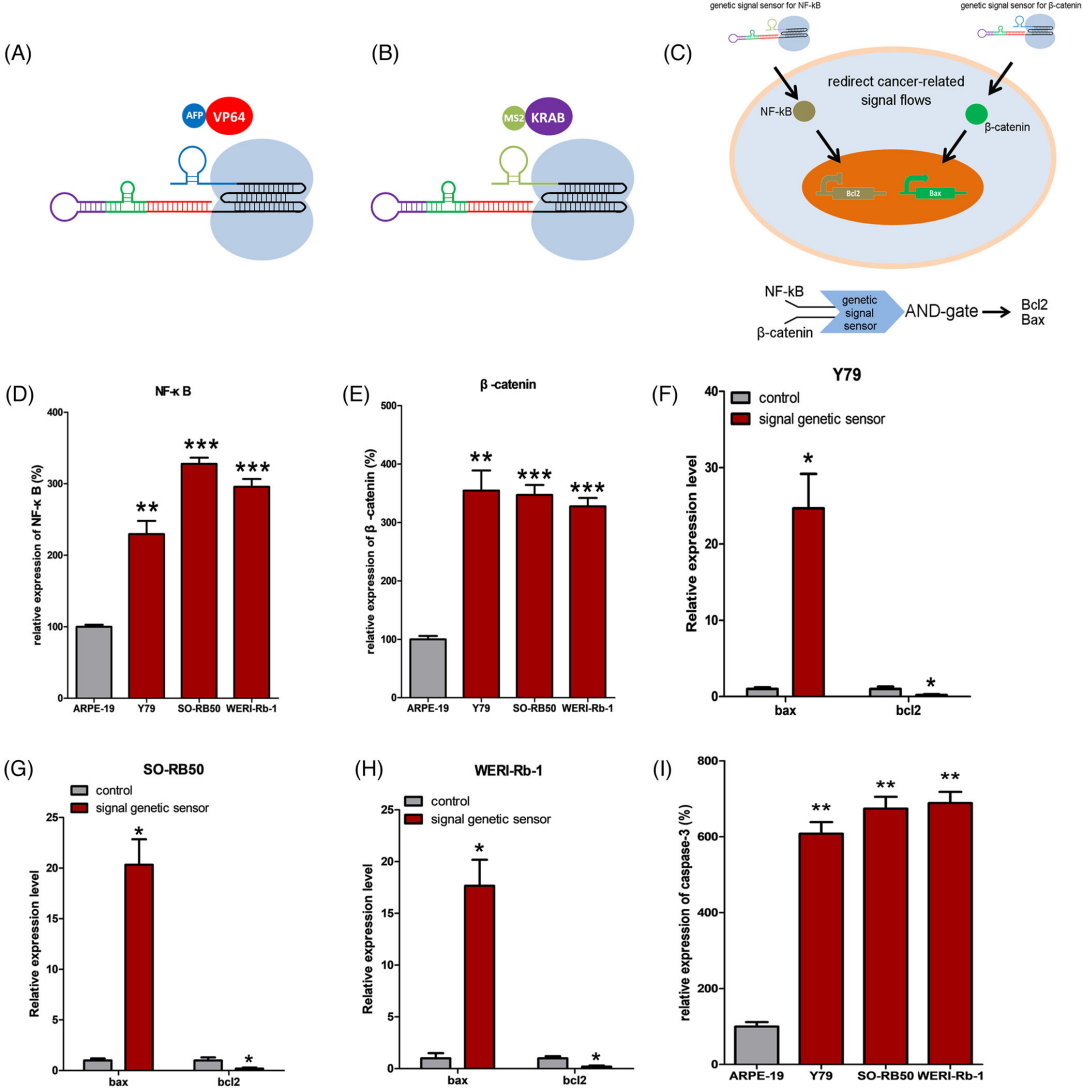
The effect of specific treatment of retinoblastomas (RBs) by the molecular‐sensor‐based artificial AND‐gate genetic circuit based on molecular sensors; we used NF‐κB and β‐catenin as input signals to construct the corresponding molecular sensors. (A) The schematic diagram of the structure of the NF‐κB sensor; we added the structure of MS2 RNA hairpins to the 3′ end of single guiding RNA (sgRNA), which recruited the MS2‐VP64 fusion protein within cells. (B) Schematic diagram of the structure of the β‐catenin sensor; we added the structure of AFP RNA hairpins to the 3′ end of sgRNA, which recruited the AFP‐KRAB fusion protein within cells. (C) The NF‐κB sensor was used to activate the expression of the *bax* gene, while the β‐catenin sensor was used to inhibit the expression of Bcl2, so as to construct an AND‐gate genetic circuit that specifically selected the RB cells from normal cells and promoted cancer cell apoptosis. (D & E) Compared with a normal cell, the expressions of NF‐κB and β‐catenin in RB cells were determined. (F, G & H) The activation effects of NF‐κB sensor on the *Bax* gene and the inhibitory effect of β‐catenin sensor on the *Bcl2* gene in RB cells; (I) compared with normal cells, the AND‐gate gene circuit had an apoptotic effect on RB cells. We repeated each experiment three times (^*^<.05, ^**^<.01, ^***^<.001)

**FIGURE 4:**
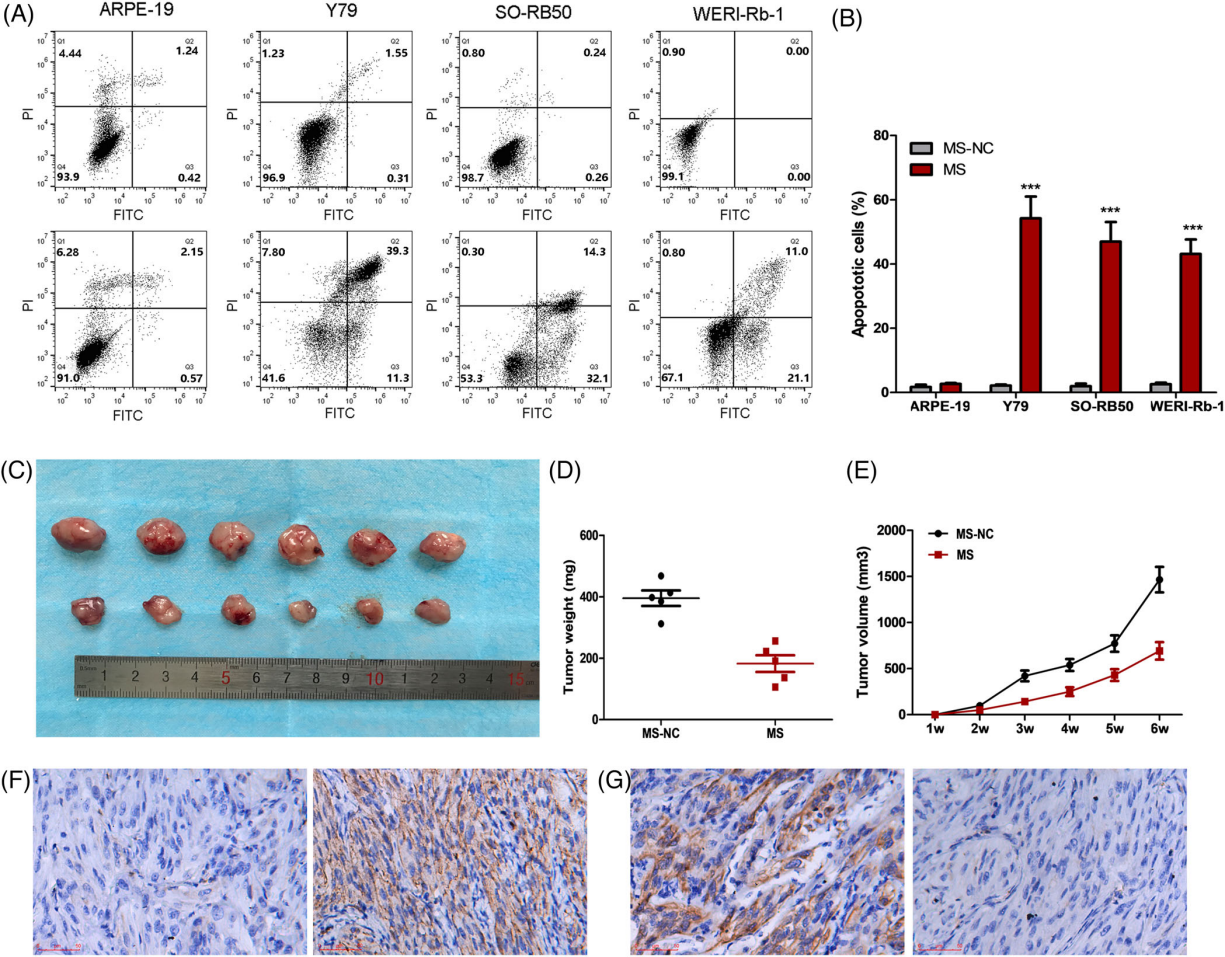
The effect of molecular sensor‐based genetic circuit promoting cell apoptosis of retinoblastoma (RB) cells in vivo; we used NF‐κB and β‐catenin as input signals to construct genetic circuits to treat RB. (A) The results of flow cytometry assay showed that artificial genetic circuits can significantly induce apoptosis of RB cells, but have little effect on normal cells. (B) Histogram of flow cytometry results, (C) tumors collected from mice, (D) tumor weights of the artificial genetic circuit and negative control treatment groups were measured and analyzed. (E) Tumor volume curves of the artificial genetic circuit and negative control treatment groups were measured and analyzed. (F) Artificial genetic circuit inhibited *bax* expression in vivo. (G) Artificial genetic circuit increased Bcl‐2 expression in vivo. We repeated each experiment three times (^*^<.05, ^**^<.01, ^***^<.001)

As a potential genetic device, molecular sensors regulated the biological behavior of RB cells by redirecting the signaling pathways of cancer cells, which may have clinical value. They were able to switch between the on state and off state by freely sensing the presence or absence of target input molecules, and they showed dose dependence of the input molecules. These characteristics all showed that these molecular sensors possessed excellent ability to control and model various systems. Although there are still some challenges in the development of clinically effective treatments for RB treatment model based on molecular sensors, this novel approach is expected to be extended to the treatment of other malignant tumors or the regulation of other cell biological processes.

## CONFLICT OF INTEREST

All authors declare that there is no conflict of interest.

## Supporting information

Supporting information
