# Supplementary material for: Synthetic molecular sensors based on CRISPR‐Cas9 redirect anticancer signal flows to treat retinoblastomas
Source: Clin Transl Med. 2021 Nov 8;11(11):e618. doi: 10.1002/ctm2.618 (PMC8574887; doi:10.1002/ctm2.618)
Supplement: Supplementary file 1 — Supporting information [file CTM2-11-e618-s001.docx]

Supplementary table 1: The cDNA sequences of sgRNAs in this study. The spacer sequences are in bold and the blocking sequences are underlined. Aptamer units in agRNAs are marked in red.

| name | relative sequences (5′-3′) |
| --- | --- |
| Wild-type sgRNA | **NNNNNNNNNNNNNNNNNNNN**GTTTTAGAGCTAGAAATAGCAAGTTAAAATAAGGCTAGTCCGTTATCAACTTGAAAAAGTGGCACCGAGTCGGTGCTTTTTTT |
| Tet-sensor  (target minimal promoter)  Tet-sensor  (target VEGF promoter)  NPM-sensor  (target P21 promoter)  NPM-sensor  (target c-myc promoter)  NPM-sensor  (target P53 promoter) | CGCGTGAGGCAGCAAAGTGCGGGTACATCCAGCTGATGAGTCCCAAATAGGACGAAAGGCCUAAAACAUACCAGAUCGCCACCCGCGCUUUAAUCUGGAGAGGUGAAGAAUACGACCACCUAGGCCTCCTGGATTCCAC**CGCACTTTGCTGCCTCACGCG**GTTTTAGAGCTAGAAATAGCAAGTTAAAATAAGGCTAGTCCGTTATCAACTTGAAAAAGTGGCACCGAGTCGGTGCTTTTTTT  GGTAGCTCGGAGGTCGTGGCGGTACATCCAGCTGATGAGTCCCAAATAGGACGAAAGGCCUAAAACAUACCAGAUCGCCACCCGCGCUUUAAUCUGGAGAGGUGAAGAAUACGACCACCUAGGCCTCCTGGATTCCAC**GCCACGACCTCCGAGCTACC**GTTTTAGAGCTAGAAATAGCAAGTTAAAATAAGGCTAGTCCGTTATCAACTTGAAAAAGTGGCACCGAGTCGGTGCTTTTTTT  GAAAGAATTCGTGACTCATCGGTACATCCAGCTGATGAGTCCCAAATAGGACGAAAAGGGAGGACGATGCGGACGGCGTCCGAGGGATGGGTATGCGGAGAGTAATTATTTCGCAGCTGTAGAGACGACGAGTCCTGGATTCCAC**GATGAGTCACGAATTCTTTC**GTTTTAGAGCTAGAAATAGCAAGTTAAAATAAGGCTAGTCCGTTATCAACTTGAAAAAGTGGCACCGAGTCGGTGCTTTTTTT  TATGTTGCCCAGGCTGGTCTGGTACATCCAGCTGATGAGTCCCAAATAGGACGAAAAGGGAGGACGATGCGGACGGCGTCCGAGGGATGGGTATGCGGAGAGTAATTATTTCGCAGCTGTAGAGACGACGAGTCCTGGATTCCAC**AGACCAGCCTGGGCAACATA**GTTTTAGAGCTAGAAATAGCAAGTTAAAATAAGGCTAGTCCGTTATCAACTTGAAAAAGTGGCACCGAGTCGGTGCTTTTTTT  CGAAATCTGATCCGGGATGCGGTACATCCAGCTGATGAGTCCCAAATAGGACGAAAAGGGAGGACGATGCGGACGGCGTCCGAGGGATGGGTATGCGGAGAGTAATTATTTCGCAGCTGTAGAGACGACGAGTCCTGGATTCCAC**GCATCCCGGATCAGATTTCG**GTTTTAGAGCTAGAAATAGCAAGTTAAAATAAGGCTAGTCCGTTATCAACTTGAAAAAGTGGCACCGAGTCGGTGCTTTTTTT |
| β-catenin-sensor  NF-κB-sensor | GGTTATCGTACCCTGTTCTCGGTACATCCAGCTGATGAGTCCCAAATAGGACGAAAAGGCCGATCTATGGACGCTATAGGCACACCGGATACTTTAACGATTGGCTTCCTGGATTCCAC**GAGAACAGGGTACGATAACC**GTTTTAGAGCTAGAAATAGCAAGTTAAAATAAGGCTAGTCCGTTATCAACTTGAAAAAGTGGCACCGAGTCGGTGC**TCATGACCC**ACACCATCAGGGT**GGGTCATGA**TTTTTTT  TCGGGAGGTTTGGTCCCGTCGGTACATCCAGCTGATGAGTCCCAAATAGGACGAAAGCATCCTGAAACTGTTTTAAGGTTGGCCGATGCTCCTGGATTCCAC**GACGGGACCAAACCTCCCGA**GTTTTAGAGCTAGAAATAGCAAGTTAAAATAAGGCTAGTCCGTTATCAACTTGAAAAAGTGGCACCGAGTCGGTGCGCCC**GGTCCCGTC**AGAGCGGAAGCGTGCTGGGCTCCGAACAGCGGAAGGTGGTTCGAAGCTGGGGCTTTGGACATAA**GACGGGACC**TTTTTTT |

Supplementary table 2: The cDNA sequences of the aptamer mutants used in negative sgRNAs.

| name | relative sequences (5′-3′) |
| --- | --- |
| Tetracycline aptamer  β-catenin aptamer  NF-κB aptamer  NPM aptamer | AAACATACCAGATTGACGCAATACCTTTAATCTGGAGAGGT  GCCTGGTATAATCACC  TATGGAATAGCGCAATACAAATTGCGATTTAAC  GAAGCTTACAAGAAGGACAGCACGAATGGGGAACATAGCC  GCTTACAACATTTGTGTAAGGGTAGTGGGGATGGCTCCGCT  CA  AGGGAGGACGGCATAAGTAACGTCCGAGGGATGGGTATGC |

Supplementary table 3: Sequence of mini-promoter

| name | relative sequences (5′-3′) |
| --- | --- |
| mini-promoter | CGCACTTTGCTGCCTCACGCGCGCACTTTGCTGCCTCACGCGCGCACTTTGCTGCCTCACGCGCGCACTTTGCTGCCTCACGCG**TATATAA**GCCAGCTTTGACCGCGTACGAACGAGATCGC |
